# Influence of Anecdotes of IVF Success on Treatment Decision Making: An Online Randomized Controlled Trial

**DOI:** 10.1177/0272989X251367783

**Published:** 2025-09-29

**Authors:** Verity Chadwick, Micah B. Goldwater, Tom van Laer, Jenna Smith, Erin Cvejic, Kirsten J. McCaffery, Tessa Copp

**Affiliations:** Women’s and Babies, Royal Prince Alfred Hospital, Camperdown, NSW, Australia; School of Psychology, Faculty of Science, The University of Sydney, NSW, Australia; The University of Sydney Business School, The University of Sydney, NSW, Australia; Sydney Health Literacy Lab, Faculty of Medicine and Health, School of Public Health, The University of Sydney, NSW, Australia; Sydney Health Literacy Lab, Faculty of Medicine and Health, School of Public Health, The University of Sydney, NSW, Australia; Sydney Health Literacy Lab, Faculty of Medicine and Health, School of Public Health, The University of Sydney, NSW, Australia; Sydney Health Literacy Lab, Faculty of Medicine and Health, School of Public Health, The University of Sydney, NSW, Australia

**Keywords:** assisted reproduction, infertility, medical decision making, narratives

## Abstract

**Background:**

Although in vitro fertilization (IVF) has enhanced fertility opportunities for many people, it also comes with considerable burden. Concerns have been raised about patients holding unrealistic expectations and continuing treatment indefinitely. This study aimed to investigate whether anecdotes of IVF success affect hypothetical intentions to continue treatment despite very low chances of success.

**Design:**

Online randomized controlled trial with a parallel 3-arm design, conducted in May 2022. After viewing a clinical vignette depicting 6 unsuccessful IVF cycles with less than 5% chance of subsequent treatment success, 606 females aged 18 to 45 years in Australia were randomized to receive either 1) an anecdote of IVF success despite limited chances, 2) the anecdote of success and an anecdote of failure, or 3) no anecdote. Outcomes were intention to undergo another IVF cycle, worry, likelihood of success, and narrative transportation.

**Results:**

There was a main effect of anecdote condition on intention to have another IVF cycle, with participants randomized to the positive and negative anecdote having higher intention than those given no additional information (mean difference = 0.65, 95% confidence interval [CI] = 0.12–1.18, *P* = 0.017). There were no differences between conditions regarding worry, likelihood of success, or narrative transportation. In adjusted analyses accounting for prior IVF experience, the main effect of anecdotes on intention was no longer statistically significant. Those with prior IVF experience reported a statistically higher likelihood of success and narrative transportation than those without prior IVF experience (mean difference [MD] = 34.28, 95% CI = 27.26–41.30, *P* < 0.001, and MD = 1.35, 95% CI = 0.96–1.74, *P* < 0.001, respectively).

**Conclusion:**

Hearing anecdotes may encourage continuation of IVF despite extremely low chances of success. These findings, along with our sample’s overestimation of IVF success, illustrate the importance of frequent and frank discussions about expected treatment outcomes.

**Trial registration:**

ACTRN12622000576729.

**Highlights:**

- The presence of IVF anecdotes increased the intention to undergo another IVF cycle despite extremely low chances of success.
- Balancing an anecdote of success with an anecdote of failure had no attenuating effect on intention.
- IVF providers should be wary of the potential impact of success stories on patients’ decision making.
- In the vignette depicting overuse of IVF, participants with previous IVF experience greatly overestimated the likelihood of success with another IVF cycle, supporting previous research finding that patients often have unrealistically high expectations about their own chance of success.

## Introduction

Although in vitro fertilization (IVF) has transformed the treatment of infertility, success rates of IVF remain modest, with only 18.2% of all initiated 2022 advanced reproductive treatments in Australia and New Zealand resulting in a live birth.^1^ However, the public often holds unrealistic expectations about IVF success rates.^2–4^ The optimal number (in terms of being cost and clinically effective) of IVF cycles is typically considered to be 3 complete cycles,^5^ with cumulative success rates of IVF showing little chance of pregnancy after approximately the fifth cycle of treatment, regardless of a woman’s age.^6^ Despite low likelihood of success, some couples continue for many additional cycles.^7^ Given the substantial physical, psychological, and financial burden of IVF,^8^ concerns have been raised about the potential harms of patients holding unrealistic expectations and continuing to pursue treatment indefinitely.^9^ Individuals experiencing infertility are also in a vulnerable position due to their strong desire for parenthood,^7,10^ putting them at high risk of overtreatment.

A previous qualitative study (*N* = 22) explored women and men’s decisions to continue treatment after multiple (3 or more) unsuccessful complete cycles of IVF.^11^ It found that although participants recalled relatively accurate statistics of conceiving with another cycle in their situation (i.e., approximately 5%–10%), those who had decided to continue tended to interpret their chance of conceiving more positively.^11^ In addition, many recalled hearing anecdotes of patients having success after a very large number of cycles, noting this contributed to their decision to continue and increased uncertainty about when to stop. These anecdotes of success could play an influential role in fuelling hope and contributing to unrealistic expectations regarding IVF effectiveness. Given that the general population and patients already overestimate the abilities of IVF, it is important to further explore the potential effect of anecdotes in this context.^12^

Quantitative research has similarly found that people can better attend to emotional or relevant anecdotal information than statistical information, highlighting the powerful influence of anecdotes on medical decision making.^13–15^ For example, a meta-analysis of 61 studies found that although statistical evidence was more persuasive than anecdotal evidence overall, this was not the case when the situation had high emotional engagement (e.g., high risk of threat, involved a health issue, or was relevant to the participant).^14^ Similarly, a more recent multimethod meta-analysis on anecdotal versus statistical processing in persuasive communication found emotionally resonant written narratives can meaningfully influence intentions even when statistical odds suggest otherwise.^16^ For example, a study by Line and colleagues^17^ examined the influence of statistical summaries and anecdotes on how participants judged the efficacy of an effective but relatively unknown treatment for chronic headaches (i.e., B_12_ injections). In 3 experiments, participants either read an anecdote from someone in the trial (treatment was effective or ineffective), summary statistics about the trial, or both and then asked whether they believed the injections to be effective medical treatments.^17^ They found that reading an anecdote that the treatment was ineffective reduced participants’ beliefs in the injections, even when they received strong statistical evidence that they were effective. This study^17^ and others^13,18,19^ suggest that a single anecdote can carry substantial weight when making a medical decision.

Taken together, these findings raise concern about the potential influence of IVF anecdotes of success against the odds on deciding whether to continue IVF treatment. Along with pairing anecdotes with easily understandable statistical evidence, it has been suggested in the context of IVF^11^ and more broadly^13,17^ that balancing anecdotes of success against anecdotes of failure may reduce reliance on anecdotes or their influence in decision making. However, it is unclear whether this would be the case in this context, as it has not been empirically tested. Given the vulnerable position and multifaceted impact of undergoing multiple cycles of IVF, it would not be ethically appropriate to test this in individuals facing this difficult decision. As such, this study aimed to examine the influence of anecdotes on intention to continue IVF treatment after multiple failed cycles using vignettes in a randomized controlled design. Specifically, we hypothesized that participants randomized to an anecdote of IVF success would have higher intention to continue IVF treatment to those shown no anecdote (hypothesis 1). We also hypothesized that pairing an anecdote of IVF success with an anecdote of IVF failure would attenuate this effect, with intention lower in those shown both anecdotes compared with those shown the anecdote of IVF success only (hypothesis 2).

## Methods

### Design

This online study used a randomized parallel 3-arm design, in which the presence or absence of an anecdote was varied (1] anecdote of IVF success, 2] anecdote of IVF success and IVF failure, or 3] no anecdote), using a balanced allocation ratio. The study is reported according to the Consolidated Standards of Reporting Trials (CONSORT) reporting guideline for randomized clinical trials.^20^

### Ethics Approval

The University of Sydney Human Research Ethics Committee approved all study methods (2022/211). All participants provided their informed consent. The study was prospectively registered in the Australian New Zealand Clinical Trials Registry (ACTRN12622000576729).

### Participants and Recruitment

Eligible participants were women aged 18 to 45 years currently living in Australia, with proficiency in English. Although male partners are also involved in decisions about IVF, previous qualitative research suggests that the decision to continue is mainly the female partner’s decision, due to them being the ones who physically go through treatment.^11^ For this reason, we recruited female participants only.

Participants were recruited through Dynata, an independent online research company, from May 11 to 13, 2022. Eligible panel members were sent an e-mail inviting them to participate. Interested participants could navigate to the study landing page, where they could read the participant information statement and provide their informed consent. Participants received modest compensation for their time in the form of points, which are redeemable for gift vouchers once a certain number of points have been accrued.

### Vignette and Intervention Conditions

All participants were presented a vignette depicting a situation of overuse of IVF (defined as >5 cycles).^6^ They were asked to imagine they had undergone 6 complete unsuccessful cycles to date and were trying to decide whether to continue with treatment. The doctor in the vignette informed them that their chance of success with another cycle was less than 5%. The vignette also mentioned important factors described in prior research on the impact of IVF and reasons for treatment discontinuation, such as the financial cost of another cycle of IVF, as well as the time and emotional and physical cost of treatment.^8,21^

To test the impact of an anecdote of IVF success, and whether balancing it with an anecdote of IVF failure would attenuate any effect, participants were then randomised to either 1) an anecdote of IVF success despite limited chance of success, 2) the anecdote of success as well as an anecdote of failure, or 3) no anecdote. In addition to the multidisciplinary team (behavioral scientists and psychologists with expertise in decision making, a gynecologist registrar, and a marketing academic specializing in narrative persuasion and consumer decision making), the vignette and anecdotes were reviewed by a consumer with experience of multiple unsuccessful IVF cycles and an IVF specialist to ensure they were accurate and realistic. See Box 1 for the exact wording of the vignette.

**Box 1:** Clinical Vignette of the IVF Specialist Visit

Imagine that you have been undergoing in vitro fertilization (IVF) with your partner for the past 2 years in attempts to conceive your first child. You have had 6 unsuccessful IVF cycles to date and are now trying to decide whether to continue or stop IVF treatment. Although most of the cost is covered by Medicare, each cycle has an out-of-pocket cost of around $5,000. Each cycle takes around 4 weeks and requires you to take some time off work for appointments (e.g., ultrasounds, egg collection, embryo transfer). Treatment has also had an emotional and physical toll as well as strained your relationship with your partner. With each failed cycle, you feel like you lose a piece of yourself, but the only way to get those pieces back is by having a baby. Although this process is incredibly difficult, having a baby is something you have always wanted and imagined for yourself. You have an appointment with your fertility specialist to discuss next steps. Your doctor says: “Although IVF is a long-term process and failed attempts are part of the process, success is most likely within five to six attempts. As you have now had six cycles of IVF, your chances of success with another cycle are less than 5%.” “Deciding to continue or stop is an incredibly difficult decision” *<Participants were then randomized to 1 or none of the following:>* **[Anecdote of success]** I had a couple with six very similar cycle results to you. They were despairing that they hadn’t had a successful pregnancy yet and were trying to work out if it was worth continuing. They decided to give it another go—and got pregnant! They’ve now got baby Oliver and are so glad they kept trying.” **[Paired anecdotes of success and failure]** “I’ve had two couples with six very similar cycle results to you. They were despairing that they hadn’t had a successful pregnancy yet and were trying to work out if it was worth continuing. Both couples decided to give it another go—The first got pregnant! They’ve now got baby Oliver and are so glad they kept trying.” “But the other couple went on to have a further three cycles but sadly still had no luck. They’ve since stopped trying due to the strain it placed on their relationship and finances.” “I am happy to support you with whatever decision you decide.”

### Measures

#### Baseline measures regarding parenthood intentions and reproductive history

In order to describe the study sample in terms of their reproductive history, demographics and fertility awareness, participants answered a series of questions regarding number of children, plans to have (more) children, if they had ever experienced trouble falling pregnant, and if they would consider using IVF or had used IVF in the past. Participants were also asked six adapted items measuring prior knowledge and beliefs about when fertility declines (for both females and males), and the chance of having a live birth with IVF for different age groups (i.e. women aged 30-34 years, 35-39 years, 40-44 years, and 45 years or more).^3^

#### Outcome measures

The primary outcome was intention to continue IVF treatment, with a free-text question included to enable participants to elucidate on their response. Secondary outcomes included worry,^22,23^ perceived likelihood of success, and narrative transportation,^24^ to investigate how the presence or absence of anecdotes influenced the degree of participant transportation or immersion into the story (see Table 1 for outcome measures).

**Table 1:** Outcome Measures

Measure and Reference	Items, Scale, and Coding Instructions
Intention to continue IVF?^25,26^	Single item: “Imagining you were in this situation, would you have another cycle of IVF?” (1 = definitely will NOT to 10 = definitely will have another cycle)
Worry^22,23^	Single item: “Imagining you were in this situation, how worried would you be about your chance of conceiving?” (1 = not at all worried to 7 = extremely worried)
Perceived likelihood of success	Single item: “On a scale from 0% to 100%, how likely do you think it is that you would be successful after another cycle of IVF?” (please slide the blue dot along the scale from 0% to 100%)
Narrative transportation^24^	Seven items measured on a 7-point scale (1 = not at all to 7 = very much). Total narrative transportation calculated by averaging items 1 to 5. Items: 1. I could picture myself in the scene of the events described in the scenario 2. I was mentally involved in the scenario while reading it 3. I wanted to learn how the scenario ended 4. The scenario affected me emotionally 5. While reading the scenario I had a vivid image of the story setting 6. While reading the scenario I had a vivid image of the couple who was successful^*^ 7. While reading the scenario I had a vivid image of the couple who was unsuccessful^**^ Scale notes: *Not administered to the no-anecdote condition **Not administered to anecdote of success only condition or no-anecdote condition

### Procedure

The study was conducted online using Qualtrics survey software. After completing the eligibility screeners and baseline measures, eligible and consenting participants were shown the clinical vignette and randomly allocated to 1 of the 3 conditions. Randomization was performed by the Mersenne Twister pseudorandom number generator in Qualtrics. Participants and researchers were blinded to the condition allocation until data collection was complete. After reading the vignette, participants completed the outcome measures, followed by additional questions about demographic and health characteristics (education, state or territory of residence, postcode, country of birth and private health insurance status).

### Sample Size

A total sample size of 390 individuals (*n* = 130 per condition) was calculated to achieve 80% power to detect pairwise differences between arms of 0.38SDs (corresponding to a 1-unit difference on the intention scale) at an adjusted alpha level of 0.025. We initially oversampled by ∼20% of the required sample size to each randomized condition (*n* = 156 per condition) to ensure a suitable sample size would remain after data cleaning.

### Data Analysis

Statistical analyses were conducted using SPSS version 23 (IBM). Data were first checked for missing values, outliers, nonserious responders (bots, completion time less than one-third of the median, nonsensical or rude responses to open-ended questions). Differences between randomized conditions on outcomes of interest (intention, worry, perceived likelihood of success) were then analyzed using ANOVA (model 1), with planned pairwise comparisons between the 3 conditions. As a result of feedback from the study’s IVF consumer about her difference in attitude prior to starting IVF and after undergoing multiple failed cycles, we also planned to conduct a sensitivity analysis including past IVF use as a moderator to see how this affected the results (ANCOVA; model 2).

## Results

**Figure 1:**
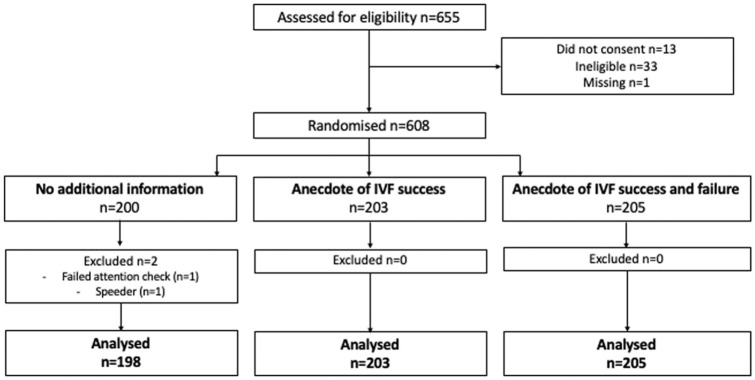
Participant flow diagram.

Of the 608 eligible participants who were randomized, 2 were excluded due to failing the attention check question or had an implausibly short completion time. This left a total of 606 participants for the final analysis (Figure 1).

### Baseline Demographics and Health Characteristics

The median age of the sample was 31 years, with most participants born in Australia (81%). Just more than one-third held a bachelor’s degree or higher (36%). Just less than one-third reported experiencing difficulties getting pregnant (27%), and 10% reported having undergone IVF. Almost half of the sample already had children (45%), and 52% were planning to have (or have more) children. Of those who had undergone IVF to conceive (*n* = 63), 75% had children.

Regarding prior knowledge of fertility and IVF success rates, fewer than one-third (28%) of participants correctly identified that female fertility markedly declined at 35 to 39 y of age, and only 7% correctly identified male fertility markedly declined at 45 to 49 y. The vast majority of participants overestimated the likelihood of having a baby from an IVF cycle, with only 9% correctly identifying (or underestimating) the chances for a woman aged 30 to 34 y (i.e., up to 24% chance per cycle), 9% for a woman aged 35 to 39 y (up to 14%), 11% for a woman 40 to 44 years (up to 9%), and 18% for a woman 45 y or older (up to 5% per cycle). Demographic and baseline characteristics by randomized condition are reported in Table 2.

**Table 2:** Demographic and Baseline Characteristics by Randomized Condition (*N* = 606)

Variable	No Anecdote (Control) (*n* = 198)	Anecdote of Success (*n* = 203)	Anecdote of Success and Failure (*n* = 205)
	*n* (%)	*n* (%)	*n* (%)
Age (y)			
18–25	64 (32)	75 (37)	67 (33)
26–35	77 (39)	63 (31)	70 (34)
36–45	57 (29)	65 (32)	68 (33)
Born in Australia			
Yes	161 (81)	164 (81)	168 (82)
No	37 (19)	39 (19)	37 (18)
Private health insurance			
Yes	113 (57)	108 (53)	120 (59)
No	85 (43)	95 (47)	85 (42)
Education (highest achieved)			
High school or below	61 (31)	63 (31)	68 (33)
Diploma or certificate	67 (34)	71 (35)	60 (29)
Bachelor degree or above	70 (35)	69 (34)	77 (38)
Have children already			
Yes	98 (49)	86 (42)	91 (44)
No	100 (51)	117 (58)	114 (56)
Plans to have (more) children			
Yes	101 (51)	108 (53)	107 (52)
No	74 (37)	61 (30)	62 (30)
Unsure	23 (12)	34 (17)	36 (18)
Importance of children			
Not very/not important	32 (16)	33 (16)	19 (9)
Neutral	25 (13)	40 (20)	47 (23)
Important/very important	84 (42)	83 (41)	96 (47)
I already have children	57 (29)	47 (23)	43 (21)
Experienced difficulty getting pregnant			
Yes	45 (23)	58 (29)	58 (28)
No	153 (77)	145 (71)	147 (72)
Would consider IVF if had difficulty conceiving			
Yes	121 (61)	125 (62)	122 (60)
No	36 (18)	37 (18)	31 (15)
Unsure	41 (21)	41 (20)	52 (25)
Previously had IVF			
Yes	13 (7)	24 (12)	26 (13)
No	185 (93)	179 (88)	179 (87)
Knowledge of decline in female fertility			
<35 y	51 (26)	49 (24)	52 (25)
35–39 y^a^	63 (32)	50 (25)	57 (28)
≥40 y	61 (31)	63 (31)	70 (34)
Do not know/age does not matter	23 (12)	41 (20)	26 (13)
Knowledge of decline in male fertility			
<45 y	63 (32)	59 (29)	60 (29)
45-49 y^a^	18 (9)	8 (4)	19 (9)
≥50 y	45 (23)	45 (22)	41 (20)
Do not know/age does not matter	72 (36)	91 (45)	85 (42)
Correct response for chance of having a baby after 1 IVF cycle^b^			
For women 30–34 y (20%–24%)	17 (9)	21 (10)	15 (7)
For women 35–39 y (10%–14%)	16 (8)	20 (10)	16 (8)
For women 40–44 years (5%–9%)	21 (11)	26 (13)	20 (10)
For women >44 y (0%–5%)	33 (17)	37 (18)	38 (19)

IVF, in vitro fertilization.

a Correct response.

b Includes those who stated the correct and lower percentages (underestimated). Percentages are of proportion who indicated the correct response for each age group. See Supplementary Table 1 for all data on this measure.

### Primary Outcome

#### Intention to have another cycle of IVF

There was a main effect of anecdote condition on intention to have another cycle of IVF, *F*(2, 603) = 3.09, *P* = 0.046, partial eta squared = 0.010. Planned pairwise comparisons revealed that those shown the anecdotes of success and failure had statistically higher intention compared with those who received no additional information (mean difference [MD] = 0.65, 95% confidence interval [CI] = 0.12–1.18, *P* = 0.017). No other pairwise comparisons were statistically significant (stats v. success, *P* = 0.073; success v. success and failure, *P* = 0.55).

After conducting sensitivity analyses including past IVF use as a moderator, there was a main effect of IVF experience on intention. Those with prior IVF experience had a statistically higher intention to have another IVF cycle as compared with those with no IVF experience (Mean (M) = 7.95 v. 5.35, MD = 2.60, 95% CI = 1.88–3.31, *F*[1, 600] = 51.37, *P* < 0.001). The main effect of anecdote condition, however, was no longer significant (*P* = 0.62). The previous simple effect of higher intention for those shown the anecdote of success and failure compared with those given no additional information remained significant only for those with no previous IVF experience (MD = 0.55, 95% CI = 0.01–1.09, *P* = 0.046). There was no interaction effect between IVF experience and condition on intention (*P* = 0.28). See Table 3 for means by condition and past IVF experience for all outcomes.

**Table 3:** Means (SEs) for Outcomes by Randomized Condition, Stratified by Past IVF Experience

Outcome	No Anecdote (*n* = 198)	Anecdote of Success (*n* = 203)	Anecdote of Success and Failure (*n* = 205)
	Mean (SE)	Mean (SE)	Mean (SE)
Intention to have another cycle of IVF^a^	5.23 (0.19)	5.71 (0.19)	5.87 (0.19)
No IVF experience	5.01 (0.19)	5.49 (0.20)	5.55 (0.20)
Prior IVF experience	8.39 (0.72)	7.38 (0.53)	8.08 (0.51)
Worry^b^	5.18 (0.13)	5.32 (0.13)	5.23 (0.11)
No IVF experience	5.20 (0.13)	5.24 (0.13)	5.18 (0.13)
Prior IVF experience	5.00 (0.49)	5.96 (0.36)	5.58 (0.35)
Perceived likelihood of success^c^	37.28 (1.90)	38.83 (2.01)	41.70 (1.90)
No IVF experience	34.15 (1.89)	35.27 (1.92)	38.54 (1.92)
Prior IVF experience	81.92 (7.14)	65.42 (5.25)	63.46 (5.05)
Narrative transportation^d^	4.35 (0.11)	4.57 (0.11)	4.70 (0.09)
No IVF experience	4.27 (0.11)	4.37 (0.11)	4.55 (0.11)
Prior IVF experience	5.43 (0.40)	6.03 (0.29)	5.78 (0.28)

IVF, in vitro fertilization; SE, standard error.

a Scale 1 to 10, higher scores = higher intention.

b Scale 1 to 7, higher scores = higher worry.

c Scale 1 to 100, higher scores = higher likelihood of success.

d Items 1 to 5 averaged (range 1–7), higher scores = higher involvement in the story.

### Secondary Outcomes

#### Worry

There was no main effect of anecdote condition on worry, *F*(2, 603) = 0.316, *P* = 0.78, partial eta squared = 0.001. After including past IVF experience, there were no main effects of condition (*P* = 0.29) or IVF experience (*P* = 0.21) nor an interaction (*P* = 0.35; see Table 3 for means).

#### Perceived likelihood of success

**Figure 2:**
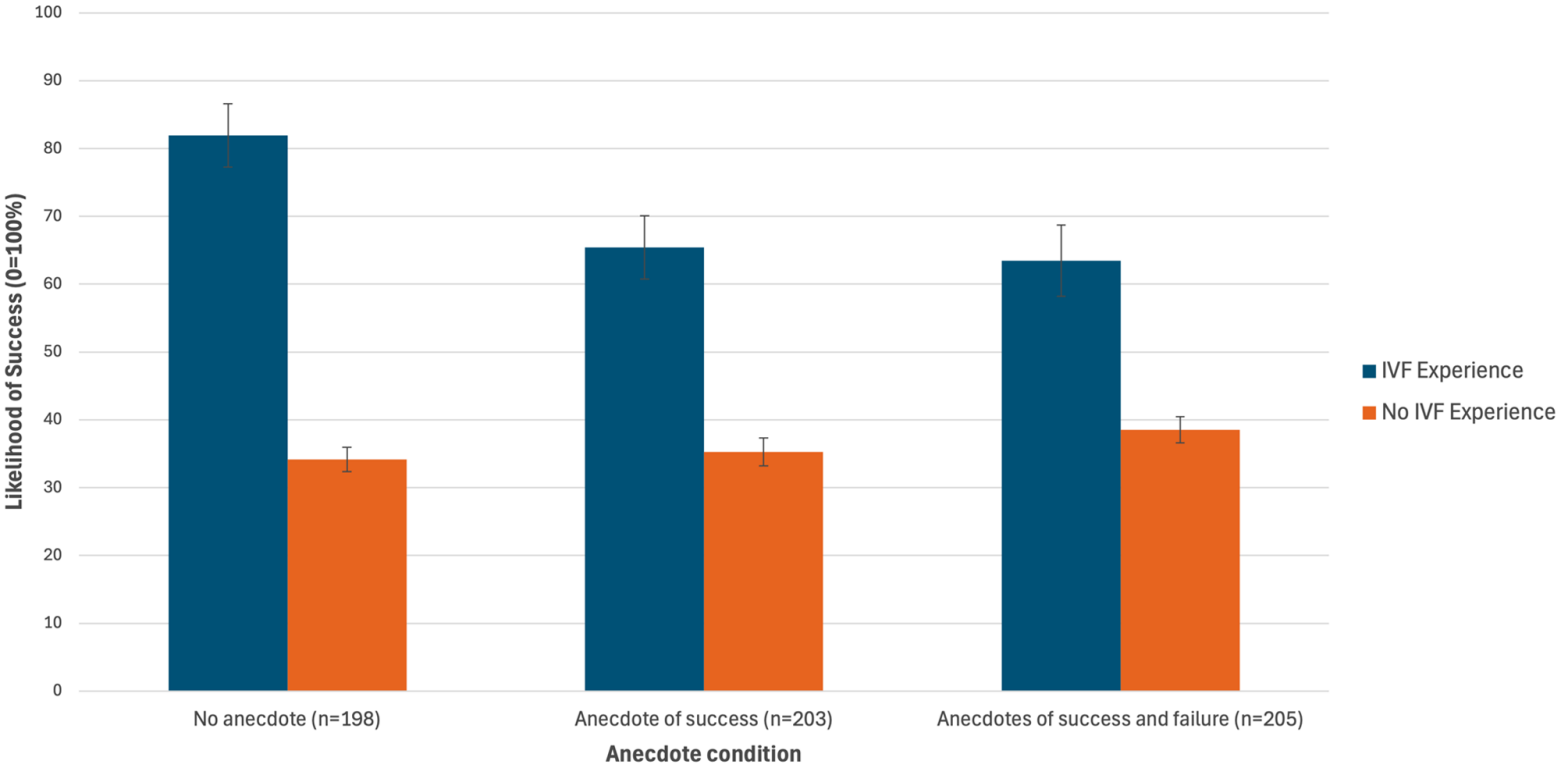
Mean perceived likelihood of success (+ standard error) with another round of in vitro fertlization (IVF) by condition and past IVF experience.

There was no main effect of anecdote condition on perceived likelihood of success, *F*(2, 603) = 1.334, *P* = 0.26, partial eta squared = 0.004. After conducting sensitivity analyses including past IVF use as a moderator, there was a main effect of IVF experience, with those with past IVF experience having statistically higher perceived likelihood of success than those with no IVF experience ( M = 70.27 v. 35.98, MD = 34.28, 95% CI = 27.26–41.30, *F*[1, 600] = 91.99, *P* < 0.001). There was also a statistically significant interaction between condition and IVF experience, *F*(2, 600) = 3.195, *P* = 0.042. Examination of pairwise comparisons showed that for those with past IVF experience, those shown the anecdotes of success and failure had a statistically lower perceived likelihood of success than those given no additional information (MD = 18.46, 95% CI = 1.29–35.63, *P* = 0.035; see Table 3 and Figure 2).

#### Narrative transportation

There was no main effect of anecdote condition on narrative transportation, *F*(2, 603) = 2.87, *P* = 0.057, partial eta squared = 0.009. After conducting sensitivity analyses including past IVF use, there was a main effect of IVF experience on narrative transportation, *F*(1, 600) = 45.73, *P* < 0.001, with those with prior IVF experience having statistically higher transportation than those with no IVF experience ( M = 5.75 v. 4.40, MD = 1.35, 95% CI = 0.96–1.74; Table 3). The interaction was not significant (*P* = 0.51).

## Discussion

In this study using a vignette of multiple failed IVF cycles, the provision of positive and negative IVF anecdotes increased intention to undergo another IVF cycle, compared with those who received no anecdote. These findings suggest that hearing anecdotes may encourage patients to continue IVF treatment despite extremely low chances of success, supporting the broader literature on narratives and medical decision making and demonstrating the powerful influence of anecdotes on medical decisions.^13–15,18,27^ Although there were no other effects of anecdotes on outcomes, the inclusion of IVF experience as a moderator had a consistently strong effect with higher intention to undergo another cycle, perceived likelihood of success, and narrative transportation.

With IVF experience included in the model, intention was statistically higher only between those shown the anecdote of success and failure compared with those given no anecdote for those with no previous IVF experience. These findings emphasize the strong emotional desire for parenthood in those who have real life experience of undergoing IVF,^7^ overshadowing any influence of anecdotes in this research context. These participants’ personal experiences of undergoing IVF are undoubtedly far more influential, vivid, and detailed than the very brief, written anecdote/s presented to them via the vignette.^28^

Our findings align with previous research showing that the public and IVF patients overestimate fertility and IVF success rates.^2–4,29–31^ Although it could have been anticipated that those with past IVF experience may have more realistic expectations and awareness of the modest success rates of IVF, this group in fact grossly overestimated the likelihood of success with another cycle. Despite being informed in the scenario that their chance of conceiving with another cycle was less than 5%, those with past IVF experience indicated their perceived chance of success was 70%. This may be due to these participants having had a successful IVF experience previously. Although we did not ascertain past IVF outcomes, 75% of those with IVF experience had children. More broadly, our results match findings from recent studies showing patients have unrealistically high expectations about their own chance of success, which, along with their strong desire for parenthood, put them at risk of overtreatment.^30,31^ Qualitative interviews with 61 women older than 40 years undergoing IVF found reasons raised for overestimating their fertility included recollections of persistent messaging about preventing pregnancy from a young age, having a healthy lifestyle and family history of fertility, incorrect information from friends and physicians, and misleading media reports of pregnancies in older celebrity women.^32^ Improved public awareness about the negative impact of age on fertility and the inability of IVF to overcome age-related infertility is needed to foster more realistic expectations of IVF success.^8^ Interestingly, in the current study, the effect of anecdotes on estimation interacted with IVF experience. For those with IVF experience, the anecdotes decreased estimations of success, but these estimates were still much higher than the estimates of those without IVF experience.

Contrary to hypothesized and conflicting with a previous study finding paired positive and negative anecdotes attenuated hypothetical intentions,^13^ balancing an anecdote of success with an anecdote of failure had no attenuating effect in the current study, with intention even higher in this group than those shown an anecdote of success only. One reason for this could be that overarching stories with highs and lows are more engaging and compelling than straightforward narratives are,^33^ which would explain the higher transportation scores of participants who received paired anecdotes of success and failure. There may also be confirmation bias in which patients are looking to cultivate hope for a biological child,^34^ and hope can have the power to propel unproven treatments that have no benefit or may even be harmful to patients, to “try anything possible” to prevent future regret and obtain reproductive closure.^35^ This may also explain why participants with IVF experience had much higher intentions and perceived likelihood of success with a further round of IVF.^34^ Another potential explanation could be related to the frequency of anecdotes. For example, one study evaluating the influence of narrative information on perceived vaccination risks and intentions found that as the number of negative narratives increased, perceived risk increased and vaccination intentions decreased.^18^

This study is not without its limitations. Our study asked participants to make hypothetical treatment decisions with limited detail, and therefore our results may not be generalizable to patients making actual treatment decisions. The consistently higher scores on outcomes for participants with past IVF experience indicate that people facing this decision in real life may respond more favorably to continuing IVF, irrespective of any information and anecdotes they are exposed to. We also did not measure the outcomes of IVF treatment for those with IVF experience, which may explain their favorable attitude toward IVF. Another limitation is that anecdotes were provided by a health care provider, not friends or family, which may carry greater weight than those from health care providers. Future research could examine the impact of anecdotes by others in patients’ lives as well as the impact of multiple anecdotes on medical decisions. We also recruited female participants only. Although IVF treatment has the biggest burden on the female partner, future studies should examine the influence of anecdotes on male participants as well. Further, for different outcome measures, the effects of anecdotes seemed to vary by subgroups. For those with IVF experience, the anecdotes decreased estimates of success, but for those without IVF experience, anecdotes increased intentions to use IVF. Future research would need to purposefully sample those with prior IVF experience to more closely match the sample size of those without to make sense of these complex patterns. Although our sample was representative of the broader Australian population in terms of education and private health insurance, we did not collect demographic data on health literacy skills, racial identity, or urban location, potentially limiting the generalizability of our results.

In conclusion, these results underscore the need to consider anecdotal bias when discussing treatment expectations and underline the complex role of experiential knowledge in fertility decision making. Assisted reproductive technology providers should be cautious of using anecdotes or stories of their previous patients’ IVF outcomes when counseling patients. Given our sample greatly overestimated the abilities of IVF, these findings also stress the importance of regularly and repeatedly discussing the likelihood of IVF success^8,10,30^ and having frank discussions about stopping treatment after multiple unsuccessful cycles.^8^

## Supplemental Material

sj-docx-1-mdm-10.1177_0272989X251367783 – Supplemental material for Influence of Anecdotes of IVF Success on Treatment Decision Making: An Online Randomized Controlled Trial Supplemental material, sj-docx-1-mdm-10.1177_0272989X251367783 for Influence of Anecdotes of IVF Success on Treatment Decision Making: An Online Randomized Controlled Trial by Verity Chadwick, Micah B. Goldwater, Tom van Laer, Jenna Smith, Erin Cvejic, Kirsten J. McCaffery and Tessa Copp in Medical Decision Making
