# Supplementary material for: Influence of Anecdotes of IVF Success on Treatment Decision Making: An Online Randomized Controlled Trial
Source: Med Decis Making. 2025 Sep 29;46(1):15–25. doi: 10.1177/0272989X251367783 (PMC12705866; doi:10.1177/0272989X251367783)
Supplement: sj-docx-1-mdm-10.1177_0272989X251367783 – Supplemental material for Influence of Anecdotes of IVF Success on Treatment Decision Making: An Online Randomized Controlled Trial [file sj-docx-1-mdm-10.1177_0272989X251367783.docx]

**Supplementary Table 1.** Estimated chance of having a baby with IVF by randomised condition (N=606)

| **Variable** | **No anecdote (control)**  **(n=198)** | **Anecdote of success**  **(n=203)** | **Anecdote of success and failure**  **(n=205)** |
| --- | --- | --- | --- |
|  | **n (%)** | **n (%)** | **n (%)** |
| Chance of having a baby after 1 IVF cycle for women 30-34 years  Underestimated  Correct (20-24%)  Overestimated | 9 (5)  8 (4)  181 (91) | 14 (7)  7 (3)  182 (90) | 5 (2)  10 (5)  190 (93) |
| Chance of having a baby after 1 IVF cycle for women 35-39 years  Underestimated  Correct (10-14%)  Overestimated | 8 (4)  8 (4)  182 (92) | 9 (4)  11 (5)  183 (90) | 9 (4)  7 (3)  189 (92) |
| Chance of having a baby after 1 IVF cycle for women 40-44 years  Underestimated  Correct (5-9%)  Overestimated | 8 (4)  13 (7)  177 (89) | 9 (4)  17 (8)  177 (87) | 8 (4)  12 (6)  185 (90) |
| Chance of having a baby after 1 IVF cycle for women >44 years  Correct (0-5%)  Overestimated | 33 (17)  165 (83) | 37 (18)  166 (82) | 38 (19)  167 (82) |
